# Supplementary material for: Cross-species functional diversity within the PIN auxin efflux protein family
Source: eLife. 2017 Oct 24;6:e31804. doi: 10.7554/eLife.31804 (PMC5655145; doi:10.7554/eLife.31804)
Supplement: Transparent reporting form [file elife-31804-transrepform.docx]

***eLife’s* transparent reporting form**

We encourage authors to provide detailed information *within their submission* to facilitate the interpretation and replication of experiments. Authors can upload supporting documentation to indicate the use of appropriate reporting guidelines for health-related research (see [EQUATOR Network](http://www.equator-network.org/%20)), life science research (see the [BioSharing Information Resource](https://biosharing.org/)), or the [ARRIVE guidelines](http://www.plosbiology.org/article/info:doi/10.1371/journal.pbio.1000412) for reporting work involving animal research. Where applicable, authors should refer to any relevant reporting standards documents in this form.

If you have any questions, please consult our Journal Policies and/or contact us: [editorial@elifesciences.org](mailto:editorial@elifesciences.org).

**Sample-size estimation**

- You should state whether an appropriate sample size was computed when the study was being designed
- You should state the statistical method of sample size computation and any required assumptions
- If no explicit power analysis was used, you should describe how you decided what sample (replicate) size (number) to use

Please outline where this information can be found within the submission (e.g., sections or figure legends), or explain why this information doesn’t apply to your submission:

For Transgenics: The number of transgenic events analyzed is referred to in the text next to the specific phenomena observed in microscopy. We initially characterized all single-insertion events derived from one transformation, and selected a subset for further analysis in the T3 generation.

For Imaging: The number of samples imaged is referred to in the text next to the specific phenomena observed.

For transport assays: Sample sizes were based on previously published standard assays and are noted in Figure legends.

Phenotypic measurements: Sample sizes are noted in the legends.

**Replicates**

- You should report how often each experiment was performed
- You should include a definition of biological versus technical replication
- The data obtained should be provided and sufficient information should be provided to indicate the number of independent biological and/or technical replicates
- If you encountered any outliers, you should describe how these were handled
- Criteria for exclusion/inclusion of data should be clearly stated
- High-throughput sequence data should be uploaded before submission, with a private link for reviewers provided (these are available from both GEO and ArrayExpress)

Please outline where this information can be found within the submission (e.g., sections or figure legends), or explain why this information doesn’t apply to your submission:

For Transgenics: The number of transgenic events analyzed is referred to in the text next to the specific phenomena observed in microscopy. All lines were analyzed multiple generations as indicated in the methods.

For Imaging: The number of samples imaged is referred to in the text next to the specific phenomena observed.

For transport assays: Assays were performed two times with similar results, but only one replicate is shown because overall radioactivity tends to vary between experiments, while relative numbers do not.

**Statistical reporting**

- Statistical analysis methods should be described and justified
- Raw data should be presented in figures whenever informative to do so (typically when N per group is less than 10)
- For each experiment, you should identify the statistical tests used, exact values of N, definitions of center, methods of multiple test correction, and dispersion and precision measures (e.g., mean, median, SD, SEM, confidence intervals; and, for the major substantive results, a measure of effect size (e.g., Pearson's r, Cohen's d)
- Report exact p-values wherever possible alongside the summary statistics and 95% confidence intervals. These should be reported for all key questions and not only when the p-value is less than 0.05.

Please outline where this information can be found within the submission (e.g., sections or figure legends), or explain why this information doesn’t apply to your submission:

For transport assays: Statistics were based on previously published standard assays and are described in Figure legends.

Phenotypic measurements: Statistics were based on previously published standard assays and are described in Figure legends

(For large datasets, or papers with a very large number of statistical tests, you may upload a single table file with tests, Ns, etc., with reference to sections in the manuscript.)

**Group allocation**

- Indicate how samples were allocated into experimental groups (in the case of clinical studies, please specify allocation to treatment method); if randomization was used, please also state if restricted randomization was applied
- Indicate if masking was used during group allocation, data collection and/or data analysis

Please outline where this information can be found within the submission (e.g., sections or figure legends), or explain why this information doesn’t apply to your submission:

Groups were allocated according to genotype, as indicated in the text.

**Additional data files (“source data”)**

- We encourage you to upload relevant additional data files, such as numerical data that are represented as a graph in a figure, or as a summary table
- Where provided, these should be in the most useful format, and they can be uploaded as “Source data” files linked to a main figure or table
- Include model definition files including the full list of parameters used
- Include code used for data analysis (e.g., R, MatLab)
- Avoid stating that data files are “available upon request”

Please indicate the figures or tables for which source data files have been provided:

Figure 1 - Source Data 1 - Spikelet and Floret Counts.txt

Figure 1 - Source Data 2 - DII quantification

Figure 2 - Source Data 1 - Internode lengths

Figure 2 - Source Data 2 - pin1b-1 complementation.txt

Figure 4 - Source Data 1 - Radioactive Counts Per Minute.txt

Figure 4 - Source Data 2 - Floral Organ Numbers

Figure 8 - Source Data 1 - Radioactive Counts Per Minute.txt

Figure 8 - Source Data 2 - Stem Cross-Sectional Area Sq mm.txt

Figure 9 - Source Data 1 - PIN1 clade alignement.fasta
